# Genes expressed at low levels raise false discovery rates in RNA samples contaminated with genomic DNA

**DOI:** 10.1186/s12864-022-08785-1

**Published:** 2022-08-03

**Authors:** Xiangnan Li, Peipei Zhang, Haijian Wang, Ying Yu

**Affiliations:** 1 Ministry of Education Key Laboratory of Contemporary Anthropology and Department of Anthropology and Human Genetics, School of Life Sciences, Fudan University, Shanghai, China; 2 State Key Laboratory of Genetic Engineering, School of Life Sciences and Human Phenome Institute, Fudan University, Shanghai, China; 3 Shanghai Pudong Hospital, Ministry of Education Key Laboratory of Contemporary Anthropology and Department of Anthropology and Human Genetics, School of Life Sciences, Fudan University, Shanghai, China; 4 Human Phenome Institute, Fudan University, Shanghai, China

**Keywords:** Genomic DNA Contamination, RNA-seq, False Discoveries

## Abstract

**Background:**

RNA preparations contaminated with genomic DNA (gDNA) are frequently disregarded by RNA-seq studies. Such contamination may generate false results; however, their effect on the outcomes of RNA-seq analyses is unknown. To address this gap in our knowledge, here we added different concentrations of gDNA to total RNA preparations and subjected them to RNA-seq analysis.

**Results:**

We found that the contaminating gDNA altered the quantification of transcripts at relatively high concentrations. Differentially expressed genes (DEGs) resulting from gDNA contamination may therefore contribute to higher rates of false enrichment of pathways compared with analogous samples lacking numerous DEGs. A strategy was developed to correct gene expression levels in gDNA-contaminated RNA samples, which assessed the magnitude of contamination to improve the reliability of the results.

**Conclusions:**

Our study indicates that caution must be exercised when interpreting results associated with low-abundance transcripts. The data provided here will likely serve as a valuable resource to evaluate the influence of gDNA contamination on RNA-seq analysis, particularly related to the detection of putative novel gene elements.

**Supplementary Information:**

The online version contains supplementary material available at 10.1186/s12864-022-08785-1.

## Background

**Fig. 1:**
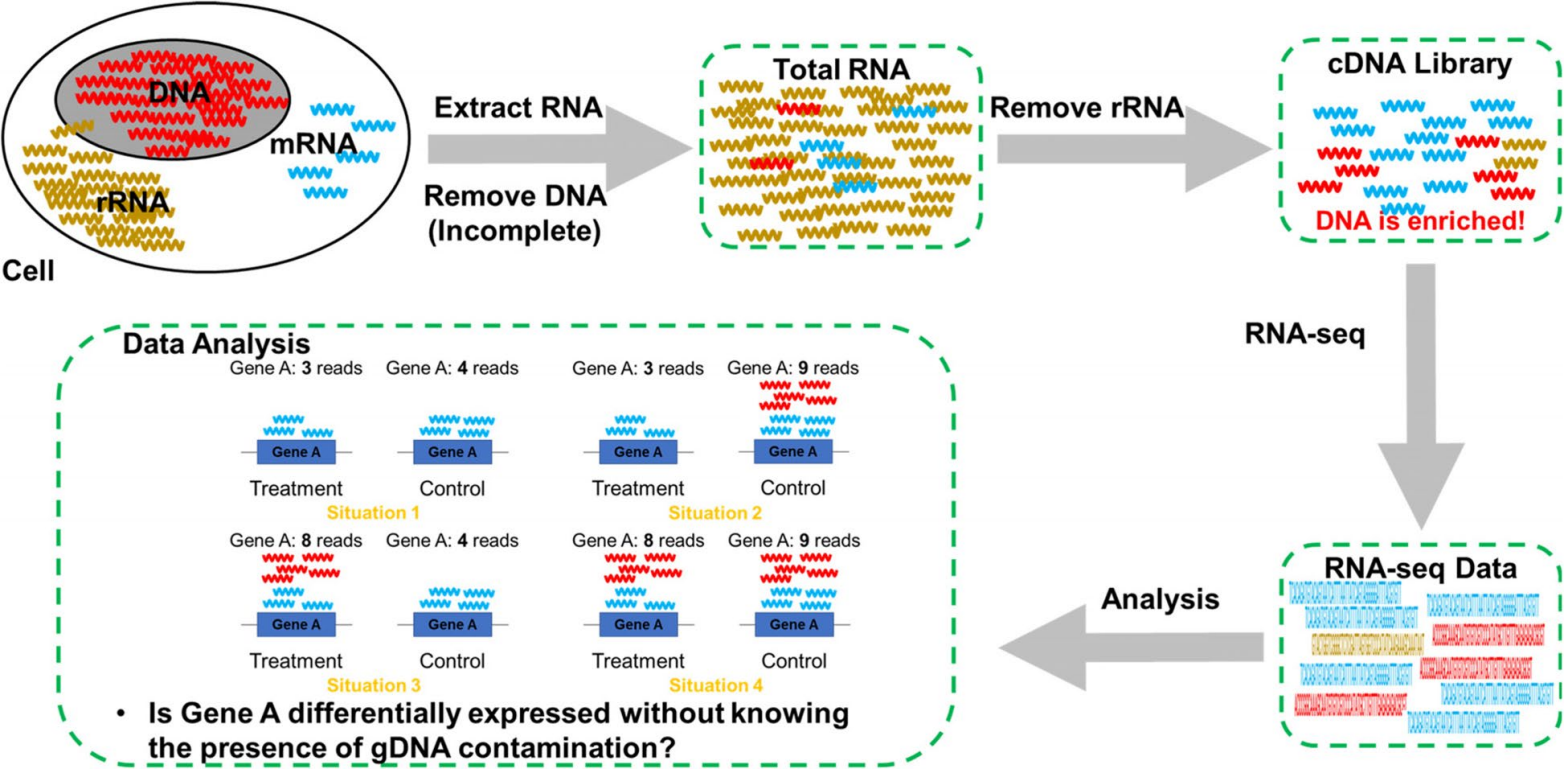
Genomic DNA contamination in RNA-seq raises concerns about the reliability of RNA-seq results. Diagram showing that genomic DNA (gDNA) contamination may affect RNA-seq results. Most RNA-seq studies focus on mRNA and/or noncoding RNA in cells or tissues, while these RNAs account for a small part of total cellular RNA. When enriching for such RNAs, gDNA will be enriched as well and eventually contaminate RNA-seq data. In extracted total RNA, the sample consists of a large amount of rRNA, small amounts of mRNAs and noncoding RNAs, and a small amount of gDNA. During library preparation, particularly using the ribosomal depletion method, most rRNA in the total RNA sample is removed, which results in high enrichment of mRNA and noncoding RNAs together with gDNA. These gDNAs will contaminate RNA-seq data and ultimately affect analyzing results, such as falsely increasing gene expression levels that may influence the DEG detection. When detecting DEGs between Treatment and Control groups, there are roughly four situations for one specific gene. Situations 1: both the Treatment and Control are not contaminated by gDNA; Situation 2: only Control is contaminated by gDNA contamination; Situation 3: only Treatment is contaminated by gDNA; Situation 4: both Treatment and Control are contaminated by gDNA. Different contaminating situations would result in different DEG detecting results for genes, e.g. gene A

Genomic DNA (gDNA) contaminates gene expression quantification techniques such as reverse transcription quantitative PCR (RT-qPCR) and microarray analysis [1, 2]. Such contamination is caused by incomplete digestion of gDNA by DNase during the extraction of total RNA [2, 3]. Library preparation for RNA-seq analysis includes digesting samples with DNase to remove contamination with gDNA that may degrade the quality of quantitative gene expression data (Fig. 1). This would introduce gDNA into RNA-seq experiment. In fact, the Sequencing Quality Control (SEQC) project found a low mapping ratio within an intergenic region, suggesting gDNA contamination of RNA-seq analyses [4]. Unfortunately, the assessment of gDNA contamination may be neglected in RNA-seq studies, although it is the focus of intense scrutiny in RT-qPCR studies [5–7]. For example, Zhou et al. proposed an extracellular RNA sequencing (exRNA-seq) strategy to determine disease status without accounting for gDNA contamination [8]. However, Verwilt et al. [6] argues that gDNA contamination may be introduced during exRNA-seq analysis, which may significantly influence the results [9]. Further, numerous studies [10–14] using RNA-seq do not report whether gDNA contamination influenced their data.

Contamination with gDNA may generate misleading data inadvertently attributed to the identification of putative novel transcribed elements through comparisons of known genomic elements. Moreover, our knowledge of completely sequenced genomes is incomplete. Therefore, claims of the detection of novel transcribed elements must be accompanied by rigorous quality control. To address this problem, Iyer et al. developed a strategy to filter gDNA reads to avoid false detection of putative novel long non-coding RNAs (lncRNAs) in RNA-seq data [15]. Further, gDNA contamination may result in inaccurate quantitation of gene expression levels that identify differentially expressed genes (DEGs).

Accurate quantitation of authentic gene expression levels using cDNAs may be significantly compromised by gDNA contamination. To address this problem, ValidPrime was developed to estimate gDNA background in RT-qPCR data [5], and several other methods are available to detect gDNA contamination in samples subjected to RT-qPCR [6, 7]. However, the influence of gDNA contamination on the quantitation of gene expression levels is unknown, which hinders the development of strategies to correct for this artifact. Further, although the incomplete digestion of gDNA by DNase is widely used to remove DNA from RNA samples, the exact concentration of residual DNA in total RNA preparations used for RNA-seq, to our knowledge, is not estimated.

To our knowledge, the effects of gDNA contamination of RNA-seq have not been systematically studied. To approach this problem, the first and critically important step is to identify genes whose expression levels are readily influenced by gDNA and to address the consequences of artifactual data. Moreover, the residual gDNA concentration must be determined, which will contribute to implementing a correction strategy. To this end, we designed an RNA-seq experiment employing different gDNA concentrations added to samples of total RNAs used to prepare libraries. We employed frequently used methods to prepare libraries for RNA-seq as follows: enrichment of polyadenylated transcripts (Poly (A) Selection) and depletion of ribosomal RNA (Ribo-Zero). Here we show that low-abundance transcripts account for inaccuracies in RNA-seq data. We therefore determined the residual gDNA concentrations and propose a data-correction strategy. The data presented here may serve as a valuable resource to evaluate the effects of gDNA contamination on the authenticity of detection of putative novel genetic elements.

## Results

### Study design

**Fig. 2:**
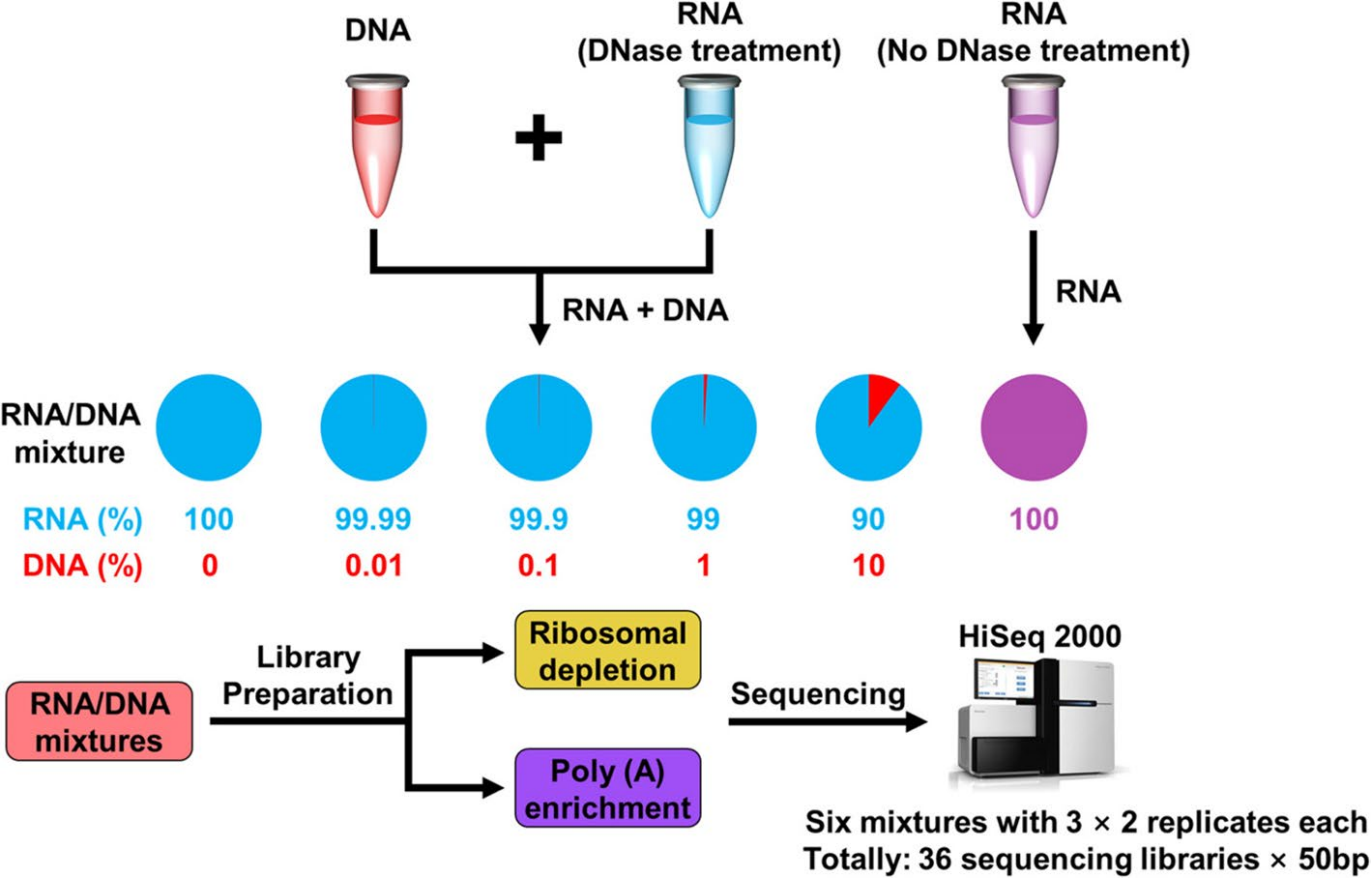
Study design. Here we aimed to investigate and reduce the influence of gDNA contamination on gene expression. Total RNA and gDNA were extracted from a human HapMap lymphoblast cell line, and total RNA was divided into two groups: one treated with DNase and the other not treated with DNase. The gDNA was added to the DNase-treated RNA to achieve concentrations of 0% to 10%. These RNA/DNA mixtures and the non-DNase-treated RNA were prepared to construct the RNA-seq libraries using the Ribo-Zero and Poly (**A**) Selection methods. Each treatment was performed in triplicate, and 36 libraries were prepared. Sequencing data (50-bp reads) were generated using an Illumina HiSeq2000

We designed an RNA-seq experiment in which we added different gDNA concentrations to total RNA for Poly (A) Selection and Ribo-Zero used to prepare the libraries (Fig. 2). Briefly, gDNA and total RNA were extracted from human HapMap lymphoblast cell lines. Total RNA was divided into DNase treatment or no treatment groups. Different amounts of gDNA were then added to DNase-treated RNA to prepare solutions ranging from 0 to 10% gDNA. These RNA/DNA mixtures together with RNA without DNase treatment were prepared for Poly (A) Selection and Ribo-Zero sequencing libraries (three replicates per mixture). The sequencing libraries (*n* = 36) were harvested, and 50-bp sequences were determined using an Illumina HiSeq 2000.

### A small amount of residual DNA in total RNA after DNase digestion

A simple linear regression model was used to fit the predicted mapping ratio within the intergenic region according to the gDNA concentration. This analysis estimated the residual DNA contamination in total RNA after DNA digestion. Approximately 1.8% of residual gDNA contamination was estimated. There was not a significant association of Poly (A) Selection between the mapping ratio and gDNA concentration (See Supplementary Table S1, Additional File 1). However, the intercept term (referred to as ***α · cDNA***_***IR_PA***_ in the Methods section) was statistically significant and therefore used to estimate the concentration of cDNAs of unannotated RNA transcripts. A significant regression equation was found for Ribo-Zero (F(1,13) = 241.6, *p* < 0.001, R^2^ = 0.949) (See Supplementary Table S2, Additional File 1). After estimating the cDNA concentrations of unannotated RNA transcripts, the fitted regression model was represented by the equation as follows:

$${{\varvec{m}}{\varvec{a}}{\varvec{p}}{\varvec{p}}{\varvec{i}}{\varvec{n}}{\varvec{g}}\_{\varvec{r}}{\varvec{a}}{\varvec{t}}{\varvec{i}}{\varvec{o}}}_{{\varvec{I}}{\varvec{R}}\_{\varvec{R}}{\varvec{Z}}}=0.658\cdot {{\varvec{D}}{\varvec{N}}{\varvec{A}}}_{{\varvec{a}}}+0.658\cdot 0.018+0.035+{\varvec{\varepsilon}}$$

where 0.018 corresponds to the residual DNA in total RNA after DNase digestion, which indicates approximately 1.8% gDNA contamination of total RNA after DNase treatment; 0.658 corresponds to the product of ***α · c***_***RZ***_*** · p***_***IR***_, and 0.035 corresponds to the product of ***α · ***$\left(1+\frac{{{\varvec{n}}}_{{\varvec{n}}{\varvec{o}}{\varvec{n}}-{\varvec{c}}{\varvec{o}}{\varvec{d}}{\varvec{i}}{\varvec{n}}{\varvec{g}}}}{{{\varvec{n}}}_{{\varvec{c}}{\varvec{o}}{\varvec{d}}{\varvec{i}}{\varvec{n}}{\varvec{g}}}}\right)$***· cDNA***_***IR_RZ***_.

This function was used to predict gDNA contamination of Ribo-Zero libraries according to the equation as follows:

$${\varvec{g}}{\varvec{D}}{\varvec{N}}{\varvec{A}}=\frac{{{\varvec{m}}{\varvec{a}}{\varvec{p}}{\varvec{p}}{\varvec{i}}{\varvec{n}}{\varvec{g}}\_{\varvec{r}}{\varvec{a}}{\varvec{t}}{\varvec{i}}{\varvec{o}}}_{{\varvec{I}}{\varvec{R}}\_{\varvec{R}}{\varvec{Z}}}-0.035}{0.658}+{\varvec{\varepsilon}}$$

where the ***gDNA*** corresponds to total gDNA contamination of total RNA used to prepare RNA-seq libraries.

### Higher gDNA contamination affects Ribo-Zero to a greater extent than Poly (A) Selection

**Fig. 3:**
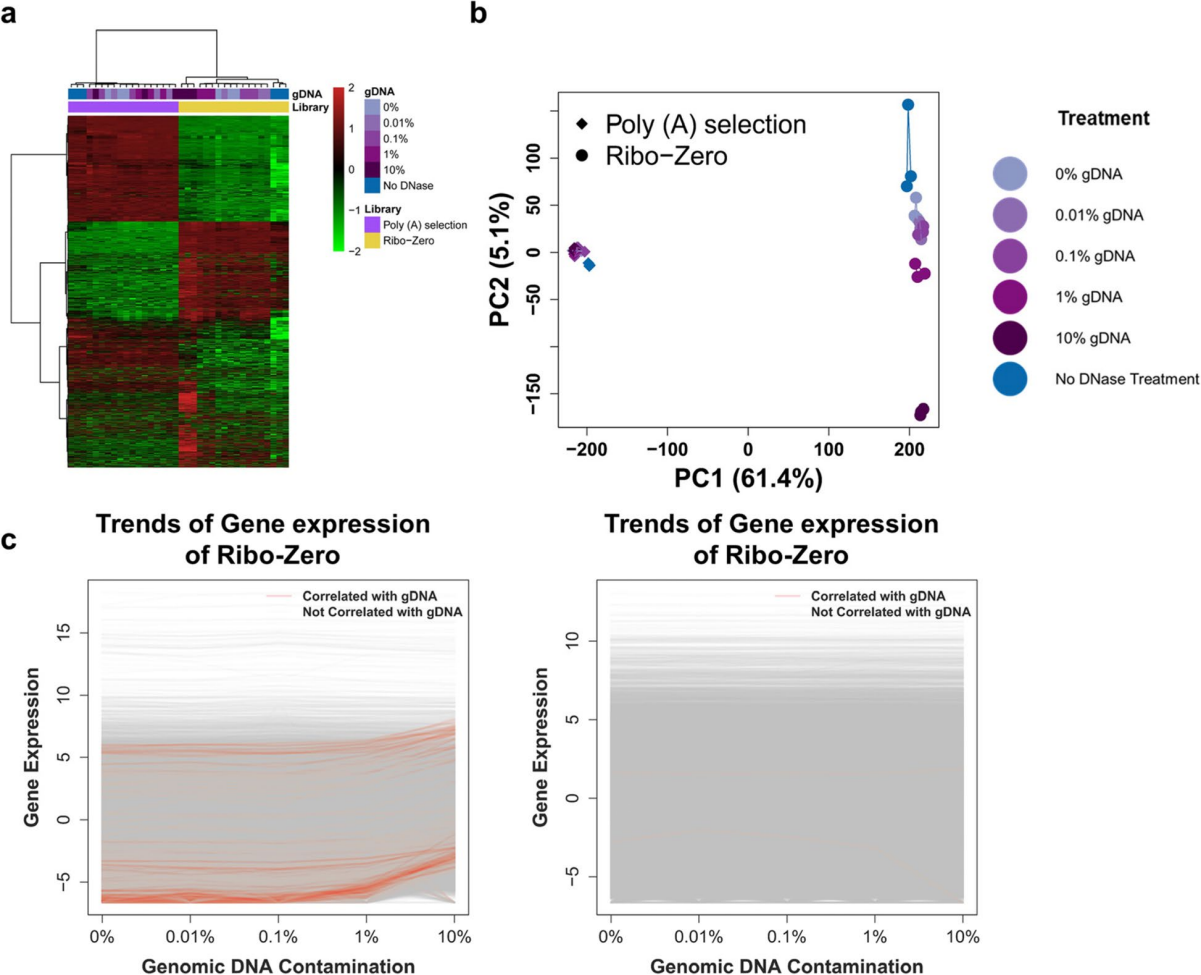
Higher gDNA contamination affects Ribo-Zero to a greater extent than Poly (**A**) Selection. **a**) Different library preparation methods clustered separately; and Poly (**A**) Selection mutually clustered, while Ribo-Zero gDNA clustered closely by the treatments. For Poly (**A**) Selection, different treatments clustered together regardless of gDNA concentrations, except for no-DNase treatment, while closely clustered by gDNA concentrations particularly at high gDNA concentrations for Ribo-Zero. **b**) PCA showed results similar to those shown in panel **a**). Different library preparation methods separately clustered. For Poly (**A**) selection, different gDNA contamination treatments tightly clustered, which reflected that gDNA exerted a small amount of influence on gene expression levels. For Ribo-Zero, different treatments tightly clustered on PC1 and sporadically on PC2, which reflects the different extents of influence of gDNA on gene expression levels. **c**) More genes were affected by gDNA, indicated by changes in their expression levels in Ribo-Zero compared with Poly (**A**) Selection. These samples were enriched in genes expressed at low expressed in Ribo-Zero (See Supplementary Figure S1, Additional File 2). Each line represents the mean expression level of one gene from three replicates at different contaminating gDNA concentrations. The x-axis represents different amounts of gDNA contamination, and the y-axis represents the gene expression value. The light red line represents gene expression levels that significantly correlated with gDNA contamination, and the gray line represents gene expression levels that did not. Left (Ribo-Zero), right (Poly (**A**) Selection)

Hierarchical cluster analysis (HCA) and principal component analysis (PCA) were used to determine the fluctuations in expression profiling caused by gDNA contamination of Poly (A) Selection and Ribo-Zero. Expression profiling showed that Ribo-Zero suffered from gDNA contamination to a significantly higher extent compared with Poly (A) Selection (Fig. 3a). For Poly (A) Selection, gDNA contamination did not significantly affect expression profiling, because most libraries mutually clustered, except those not treated with DNase. For Ribo-Zero, high gDNA levels of gDNA contamination (1% and 10%) and not treated with DNase, the libraries closely clustered. Though three replicates of 0.1% gDNA seemed clustered, they clustered with two replicates of 0.01% gDNA. The PCA and HCA results were similar for Poly (A) Selection closely clustered libraries, and Ribo-Zero libraries with 1% and 10% gDNA contamination were distinguished from the other libraries according to principal component 2 (Fig. 3b). The HCA and PCA results indicate that expression profiling using Ribo-Zero is more sensitive to gDNA contaminations compared with Poly (A) selection.

In single gene expression analysis, more genes correlated with gDNA in Ribo-Zero compared with Poly (A) Selection (510 and 2 genes for Ribo-Zero and Poly (A) Selection, respectively) (Fig. 3c and Supplementary Figure S1, Additional File 2). When we analyzed genes with expression levels that correlated to the gDNA concentrations (*p* < 0.05, two-sided, Bonferroni adjusted), we found that 94.1% that correlated with gDNA were expressed at levels < 0 (log2 FPKM (Fragments per kilobase of transcript per million read pairs)) (See Supplementary Figure S1, Additional File 2) using Ribo-Zero (at 0% gDNA contamination). The two genes that correlated with gDNA using Poly (A) Selection were expressed at values > 0 or < 0, respectively. The number of genes that correlated with gDNA support the conclusion that Ribo-Zero was more sensitive to gDNA contamination compared with Poly (A) Selection, according to the expression of a single gene.

### Genomic DNA alters the quantitation of low-abundance transcripts, leading to false-positive results using Ribo-Zero

**Fig. 4:**
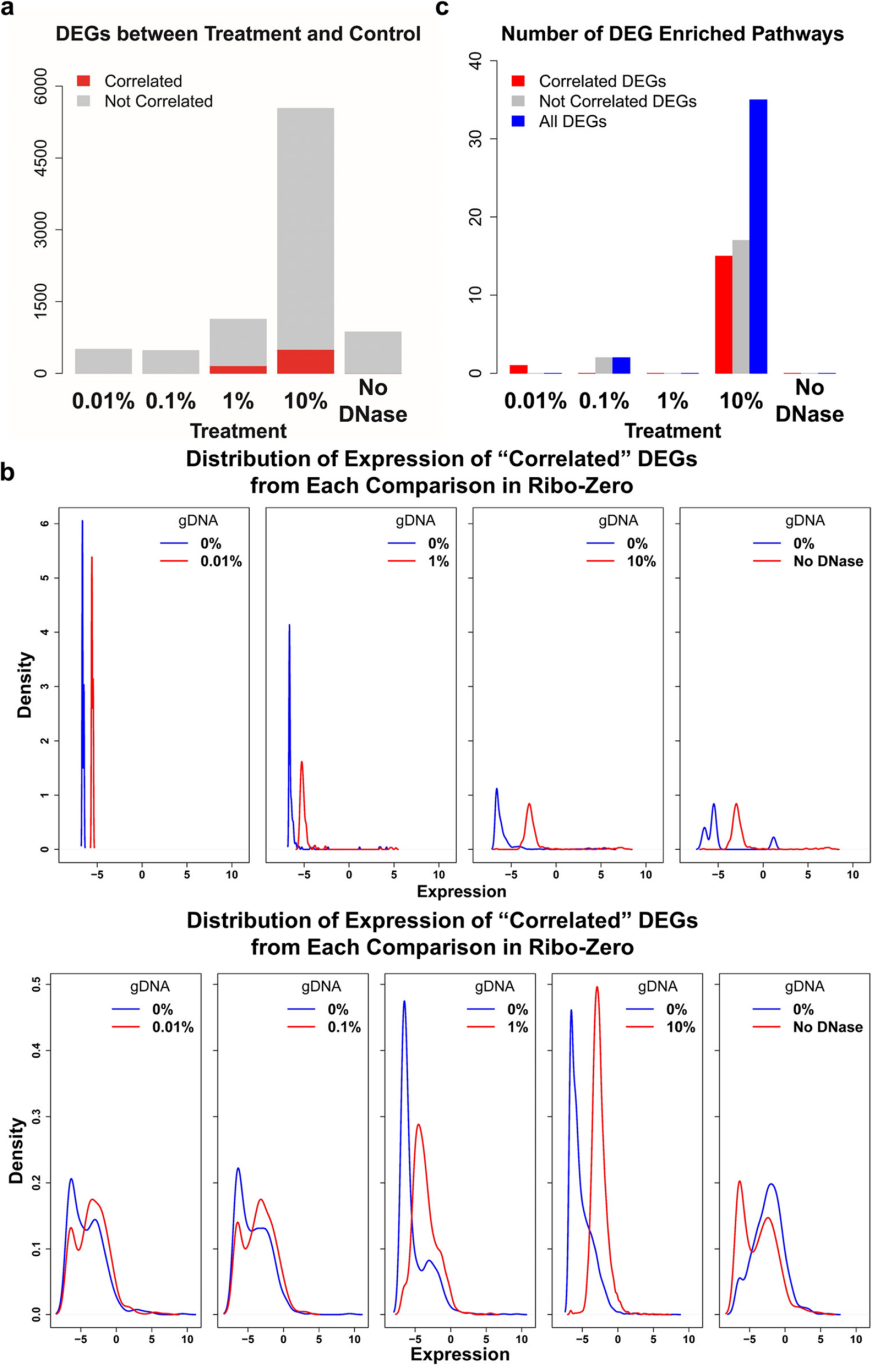
Genomic DNA alters the expression of low-abundance transcripts and leads to false results in Ribo-Zero. a) Genomic DNA significantly altered the quantitation of gene expression levels in Ribo-Zero. The bar plot shows the number of DEGs in Ribo-Zero at different concentrations of contaminating gDNA. The “Correlated” DEGs were considered genes with altered expression levels caused by gDNA contamination, and the “Not Correlated” DEGs were considered genes with altered levels caused by gDNA and/or background noise. The DEGs were detected by comparing libraries with > 0% (Treatment) and 0% (Control) gDNA. The x-axis represents different treatments; the y-axis represents the number of DEGs in each comparison (*t* test, two-sided, p < 0.05 and |log2(fold-change)|> 1). The red and gray bars represent “Correlated” and “Not Correlated” DEGs, respectively. b) The “Correlated” and “Not Correlated” DEGs were expressed at low levels in the Treatment and Control. Most “Correlated” and “Not Correlated” DEGs in Treatment and Control showed expression levels < 0. The distribution of expression levels of “Correlated” DEGs between libraries with 0.1% and 0% gDNA contamination is not displayed, because only one “Correlated” DEG was detected. The x-axis represents the expression value (log2[FPKM]); the y-axis represents density. The blue line represents Control, the red line represents Treatment. c) “Correlated” and “Not Correlated” DEGs give “false” enrichment results. The plot shows the number of enriched KEGG pathways of DEGs between Treatment and Control in Ribo-Zero. The x-axis represents different treatments; the y-axis represents the number of enriched pathways. The red, gray, and blue bars represent “Correlated”, “Not Correlated,” and all DEG-enriched pathways, respectively

DEGs were detected in libraries with > 0% gDNA (Treatment) and libraries with 0% gDNA (Control). The number of DEGs increased as the gDNA contamination increased using Ribo-Zero and were approximately constant using Poly (A) Selection (Fig. 4a and Supplementary Figure S2, Additional File 2). For Ribo-Zero, the numbers of DEGs were 504 and 477 at low gDNA concentrations (0.01% and 0.1%), respectively. When gDNA contamination was increased to 1%, the number of DEGs significantly increased to 1134; and 5533 DEGs were detected when gDNA contamination was 10%, and to 867 for libraries without DNase treatment (Fig. 4a). For Poly (A) Selection, the number of DEGs averaged 303, and for libraries without DNase treatment, 530 DEGs were detected (See Supplementary Figure S2, Additional File 2).

Although the number of DEGs increased as gDNA contamination increased, the DEGs detected using Ribo-Zero cannot be attributed to gDNA contamination simply because of background noise. Hence, the DEGs were divided according to whether one gene correlated with gDNA as follows: “Correlated” and “Not Correlated” DEGs, which represented DEGs with expression levels significantly correlated with gDNA contamination concentration (p < 0.05, two-sided, Bonferroni adjusted) and those not correlated with gDNA contamination concentration. The “Correlated” DEGs were most likely caused by gDNA contamination, and the “Not Correlated” DEGs were detected because of gDNA contamination, background noise, or both. For DEGs between libraries with 0.01% and 0% gDNA as well as those between libraries with 0.1% and 0% gDNA, there were few DEGs classified as “Correlated” DEGs (0.6% and 0.2%). For DEGs between libraries with 1% and 0% gDNA and between libraries with 10% and 0% gDNA, there were approximately 14.2% and 9.1% DEGs, respectively, classified as “Correlated” DEGs. For DEGs between libraries without DNase treatment and with 0% gDNA, 0.8% DEGs were classified as “Correlated” DEGs. Considering that the number of DEGs increased in the presence of 1% gDNA, these results suggest that when present at relatively high concentrations, gDNA contamination may alter gene quantitation.

There were low levels of DEGs attributable to gDNA and background noise (Fig. 4b). Expression levels of most “Correlated” and “Not Correlated” DEGs were > 0 (log2[FPKM]) in the Treatment and Control groups. These DEGs generated false Kyoto Encyclopedia of Genes and Genomes (KEGG) [16] enrichment results in pathway analysis (Fig. 4c). The “Correlated” DEGs were enriched in 1 and 15 pathways with 0.01% and 10% gDNA contamination, respectively; and the “Not Correlated” DEGs were enriched in 2 and 17 pathways at 0.1% and 10% gDNA contamination, respectively. When we considered “Correlated” and “Not Correlated” DEGs together, more enriched pathways were identified only at 10% gDNA contamination that 35 enriched pathways were identified. These results indicate that gDNA contamination altered the quantitation of low-abundance transcripts and led to the enrichment of false-positive pathways.

### Insignificant contribution of gDNA contributes to Pathway Enrichment Analysis

**Fig. 5:**
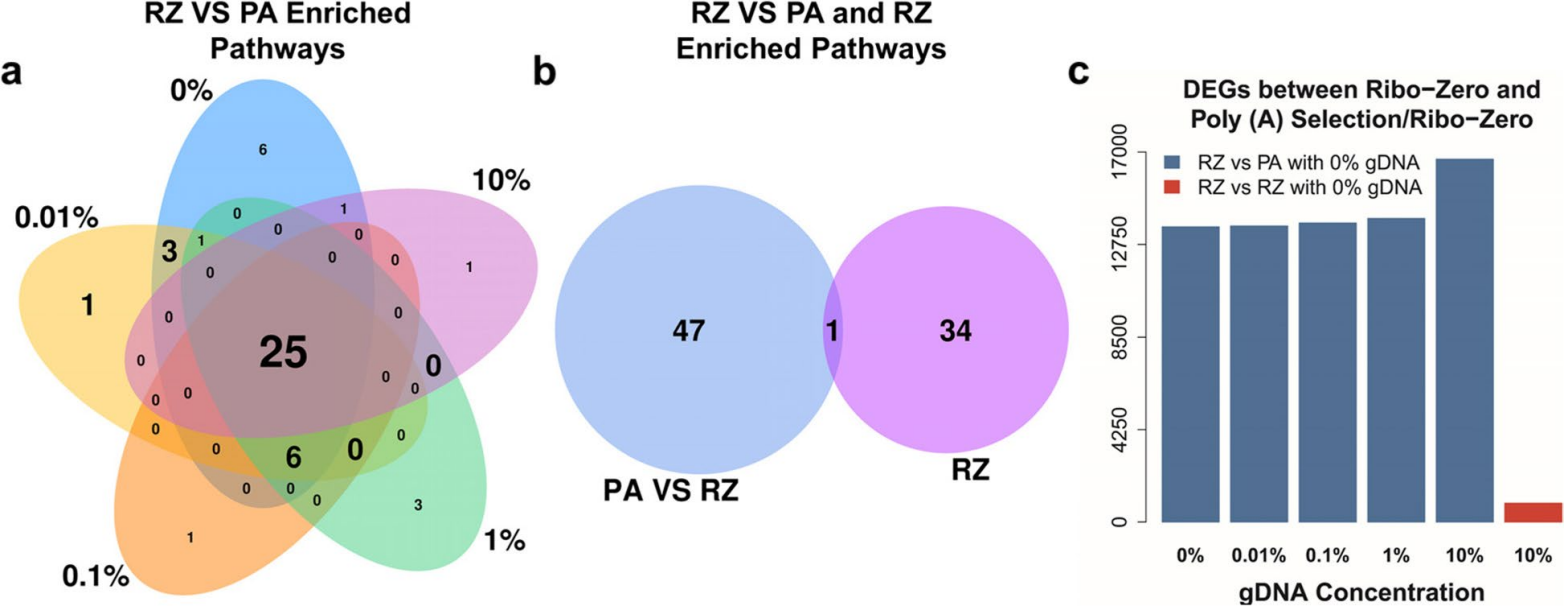
Genomic DNA contributes little to pathway enrichment analysis when comparing two distinct methods. **a**) Enriched pathways showed a large overlap regardless of gDNA concentration. The Venn diagram shows the number of DEG-enriched pathways compared with Ribo-Zero and Poly (A) Selection. Twenty-five (52.1%) of enriched pathways were shared, regardless of gDNA contamination. **b**) Most enriched pathways associated with gDNA did not appear in the comparison of Ribo-Zero and Poly (**A**) Selection. The Venn diagram shows the number of all enriched pathways in the comparison of Ribo-Zero with > 0% and 0% gDNA and Ribo-Zero and Poly (A) selection. The pathway enriched between Ribo-Zero with > 0% and 0% gDNA were considered associated with gDNA. c) Many more DEGs between Ribo-Zero and Poly (**A**) Selection than between Ribo-Zero libraries. The DEGs were detected by comparing Ribo-Zero libraries (0% to 10% gDNA) and Poly (**A**) Selection libraries (0% gDNA) and between Ribo-Zero libraries with 10% and with 0% gDNA. PA: Poly (A) Selection; RZ: Ribo-Zero

Though gDNA contamination may alter the quantitation of expression levels, particularly of low-abundance transcripts, we found that it insignificantly contributed to the pathway enrichment results when comparing two distinct samples (Fig. 5a). When we compared libraries prepared using Ribo-Zero to those with 0% gDNA prepared using Poly (A) Selection, the DEG-enriched pathways largely overlapped. There were 25 overlapping enriched pathways regardless of gDNA concentration (Fig. 5a) among 48 enriched pathways shared between Ribo-Zero and Poly (A) Selection. Further, we detected only one overlapping pathway between enriched pathways by comparing Ribo-Zero libraries to Poly (A) Selection libraries with 0% gDNA and by comparing Ribo-Zero libraries with 10% gDNA to libraries with 0% gDNA (Fig. 5b). These small overlaps may be explained by an over-abundance of DEGs (Fig. 5c). That is, too many DEGs in the background of enrichment analysis (See Supplementary Figure S3a, Additional File 2) between Ribo-Zero and Poly (A) Selection resulted in the pathways that enriched in the comparison between Ribo-Zero libraries did not enriched statistically significant. For example, there were many overlapping DEGs (See Supplementary Figure S3b, Additional File 2) between those identified through the comparison between libraries prepared using Ribo-Zero and Poly (A) Selection and DEGs from the comparison between libraries with 0% and 10% gDNA prepared using Ribo-Zero in pathway “hsa04740”. However, the pathway “hsa04740” was involved with numerous background DEGs from the former which lead to an insignificant enrichment (See Supplementary Figure S3c, Additional File 2). These results suggest that if the two groups were vastly different, the intrinsic difference between the two conditions would dilute the contribution of gDNA to pathway enrichment.

### Adjusting expression levels reduces the alteration of quantitation of expression levels using Ribo-Zero

**Fig. 6:**
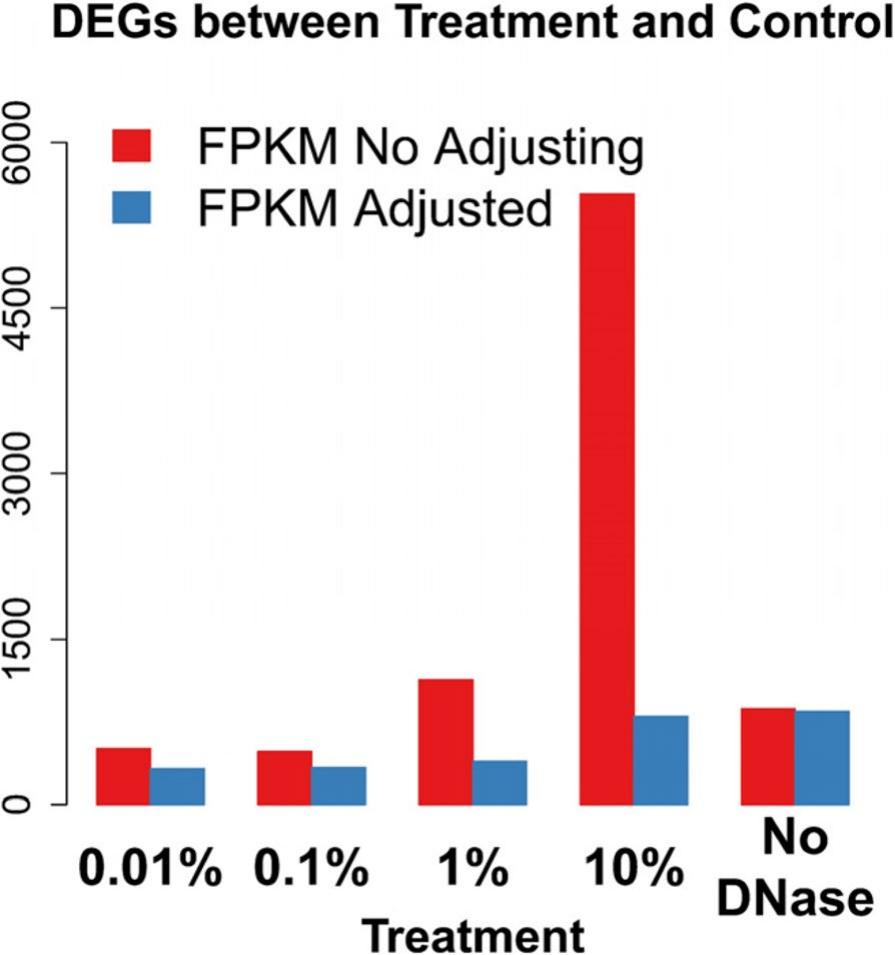
Adjusting expression levels reduces number of DEGs. The DEGs were detected by comparing libraries with > 0% (Treatment) gDNA and those with 0% (Control) gDNA for Ribo-Zero libraries. The red and blue bars represent DEGs detected before and after adjustment, respectively. The x-axis represents different treatments; the y-axis represents the number of DEGs in each comparison. (*t* test, two-sided, *p* < 0.05 and |log2(fold-change)|> 1)

Gene expression levels were adjusted by subtracting FPKM associated with gDNA from FPKM calculated using the quantitation software. The number of DEGs was largely reduced for 1% and 10% gDNA with Ribo-Zero. The number of DEGs decreased from 1134 to 333 and from 5533 to 799 in the presence of 1% and 10% gDNA, respectively (Fig. 6). However, this strategy was judged not suitable for Poly (A) Selection, because the number of DEGs increased after FPKM adjustment (See Supplementary Figure S4, Additional File 2).

## Discussion

Contamination of gene expression libraries is a common yet important problem inherent in gene quantitation technologies; however, the effects of gDNA contamination associated with RNA-seq analysis are infrequently discussed. While gDNA contamination had led to debates about doubtable results in exRNA sequencing [8, 9], it should attract more attentions. Here, we designed an experiment employing different gDNA concentrations in RNA-seq libraries to evaluate the effects of gDNA contamination on gene expression levels. We show here that contamination with gDNA altered the quantitation of low-abundance transcripts, which generated false results. These findings will serve as a valuable resource to determine the effects of gDNA contamination in studies aimed to discover novel genetic elements.

There is always a small amount of gDNA contamination in RNA-seq libraries and the extent of gDNA contamination could be estimated. Here we found that RNAs used for RNA-seq were contaminated with approximately 1.8% of gDNA after DNA digestion through a simple linear regression model. This result may have been an overestimate, because the intergenic region defined here was not sufficiently extensive, and therefore unannotated transcripts were considered gDNA contaminants. However, this finding is consistent with those of other gene quantitation methods that do not completely remove gDNA using DNase [2, 6, 7]. The linear regression model was used to estimate the gDNA contamination of one sequenced Ribo-Zero library. Contamination with gDNA is a critically important problem for cancer research, because most clinical tumor specimens are formalin-fixed, paraffin-embedded (FFPE) tissues [17] containing degraded RNA [18]. The ribosomal depletion method employed for FFPE samples might introduce more gDNA contamination. This is because the gradual fragmentation of DNA sequences in FFPE samples during storage [19, 20] makes the length of fragmented DNA sequences close to the desired length of RNA fragments, which would give the fragmented DNA sequences a high chance to be co-extracted with RNA and lead to higher DNA contamination. Indeed, we found 2.2%–7.5% gDNA contamination of Ribo-Zero libraries prepared from some FFPE samples of triple-negative breast cancer [10]. Besides, with unknown sample types, we found a higher gDNA contamination, ranging from 0.7% to 22.7%, of Ribo-Zero libraries prepared from normal human adult and human fetal tissue samples.

RNAs used to prepare libraries were contaminated with gDNA if prepared using Ribo-Zero but not Poly (A) Selection. Relatively high concentrations of gDNA contamination would cause clustering of the libraries from Ribo-Zero, whereas libraries prepare using Poly (A) Selection mutually clustered (Fig. 3a, b). This result indicates that gDNA contamination of total RNA would readily persist in a ribosomal RNA-depleted library, but not in a library enriched in polyadenylated transcripts. This conclusion is consistent with the view of gDNA contamination in RNA-seq analysis [21]. The molecular reason behind this could be attributed to the differences in target RNA capturing methods between Poly (A) Selection and Ribo-Zero. Poly (A) Selection uses oligo-T probes to capture mature mRNAs with a ~ 250 bp Poly-A tail located in the 3’ end. The gDNA fragments had a low chance to carry a 3’ Poly-A tail with ~ 250 bp, which lead to a low capturing rate by oligo-T probe. Thus, Poly (A) Selection was less prone to be contaminated by gDNA. The target RNA capturing method of Ribo-Zero is removing all rRNA from total RNA and the remaining RNAs are considered as target RNA. So any gDNA sequences in total RNA would have a relatively high chance to be captured, which led to Ribo-Zero libraries were contaminated with gDNA.

Genomic DNA contamination of total RNA is associated with gene expression. Thus, our present analysis of the expression levels of certain genes correlated with gDNA contamination (Fig. 3c). However, the alterations in expression levels of these genes were low until gDNA contamination reached 10%, indicating that gDNA influences the quantitation of expression levels when present at a relatively high concentration. Further, we found no significant difference in GC contents between these consistently altered genes and other genes (*t* test, *p* = 0.531, two-sided), indicating “GC content bias”, which indicates that regions with high GC content tend to yield more read coverage [22], and further that RNA-seq may not contribute to the increase in expression levels of these genes. Moreover, the number of DEGs increased as a function of an increase in gDNA concentrations (Fig. 4a). Though the noise associated with RNA-seq technology may increase the number of false-positive DEGs [4], the number of DEGs caused by noise was proximately equal in libraries prepared in the presence of 0.01% or 0.1% gDNA, indicating that numerous DEGs detected in libraries contaminated with 1% or 10% may be explained by contamination with gDNA.

Low-abundance transcripts identified as DEGs were most frequently associated with libraries contaminated with gDNA sequences. Though small, gDNA contamination is expected to have limited influence on analysis of gene expression data. Genes are defined as DEGs if their levels exhibit a log2 (fold-change) with an absolute value > 1 [23, 24]. For this reason, the levels of highly expressed genes must change by a relatively big amount, and difficult for them, to be detected as a DEG. Here we show (Fig. 4b) that with 10% gDNA contamination, the FPKMs of most DEGs (“Correlated” and “Not Correlated” DEGs), were < 0 (log2) in the Treatment and Control groups. If the gDNA contamination of sequenced samples was not assessed, any DEGs expressed at low levels, or novel weakly-expressed transcripts, in both comparison groups should be regarded as suspicious.

False DEG discoveries may arise from the expression of altered genes. Here we show that gDNA contamination led to the discovery of false DEGs (Fig. 4a). Although the noise intrinsic to RNA-seq analysis may also lead to the identification of false DEGs [4], the number of these DEGs may be reflected by the number of DEGs in analyses using Poly (A) Selection (See Supplementary Figure S2, Additional File 2) and Ribo-Zero, with gDNA contamination < 1%. In contrast, the number of DEGs rapidly increased in RNA samples contaminated with ≥ 1% gDNA, indicating that such DEGs were the result of gDNA contamination.

The other false discoveries were made in the DEG-enriched pathways. When we compared samples with or without gDNA contamination, many DEG-enriched pathways were identified (Fig. 4c). When the samples were distinct, such as those prepared employing libraries from Poly (A) Selection and Ribo-Zero, fewer enriched pathways were associated with gDNA concentrations. Instead, many shared pathways were identified, regardless of gDNA concentrations (Fig. 5a). These findings may be explained by the large numbers of DEGs identified using Poly (A) Selection or Ribo-Zero, which contributed differences that were not significant (e.g., *p* > 0.05) in pathway enrichment analysis (See Supplementary Figure S3c and S3d, Additional File 2). These results also indicate that pathway enrichment was sensitive to gDNA contamination when samples exhibited similar expression profiles because of the small number of DEGs (See Supplementary Figure S3c and S3d, Additional File 2). Further, in most cases, comparison of samples prepared from the same tissue did not detect a significant difference. Moreover, when experimental conditions are similar, gDNA contamination may result in the identification of falsely enriched pathways. Notably, other enrichment analyses such as Gene Ontology that only require a DEG list, would also be subject to gDNA contamination.

The false discoveries caused by gDNA contamination may be eliminated by adjusting gene expression levels. Although simply excluding genes at low levels decreases the detection of false DEGs (See Supplementary Figure S5, Additional File 2), authentic DEGs expressed at low levels may not be identified. We therefore adjusted expression levels here rather than simply eliminating low-abundance transcripts. Further, we show here that such adjustments effectively reduced the number of false DEGs in libraries prepared using Ribo-Zero (Fig. 6).

Our findings suggest that a small amount of residual gDNA contamination is present in total RNA after DNase digestion and that gDNA contamination of RNA-seq libraries will alter the quantitation of the expression levels of low-abundance transcripts, culminating in false-positive results. The linear regression model built in this study provided a way to quantitate gDNA contamination, as the assessment of gDNA contamination was not sufficiently reported in numerous RNA-seq articles [10–14, 25]. The higher gDNA contamination in Ribo-Zero compared to Poly (A) Selection suggested that When studying mRNA, for the samples with good mRNA quality, Poly-A enrichment library construction should be employed; considering the even higher gDNA contamination in RNA from FFPE samples when studying non-coding RNA, the library constriction method have to be Ribosomal depletion, however, it is better to choose cell line samples and/or FFPE samples in a short storage time. Further, the alteration of gene expression levels may be eliminated by adjusting expression levels according to the mapping ratio within the intergenic region. The present data may facilitate estimates of the contribution of gDNA contamination to gene detection, novel transcript discovery, or to the identification of the functions of unannotated RNAs.

Our study has the limitations as follows:

- 1. The limited number of concentrations skewed toward 0%. These limited concentrations may influence the estimated accuracy of linear regression; however, the trend was obvious between the intergenic region-mapping ratio and gDNA concentration, and the core finding that the expression levels of low-abundance transcripts altered by gDNA were not significantly associated with these limited concentrations.
- 2. The estimated number of unannotated transcripts in Ribo-Zero. The number of estimated unannotated transcripts in Ribo-Zero libraries may influence the estimation of residual gDNA contamination. Ideally, the unannotated transcripts in Ribo-Zero libraries should be estimated using libraries exclusively prepared using Ribo-Zero. However, it is difficult to distinguish reads from gDNA and cDNA after reverse transcription. Alternatively, we used the unannotated transcripts in Poly (A) Selection libraries to approximate the unannotated transcripts in Ribo-Zero libraries using the ratio of annotated coding genes to noncoding genes.
- 3. The generality of the defined intergenic region. The definition of an intergenic region may influence the quantitation of gDNA contamination. The gene expression data are tissue/cell type-specific and may therefore lose accuracy for estimating the expression caused by gDNA contamination of tissues/cells other than blastoma cells. Though several assumptions were made here, most are basic to RNA-seq analysis.

To further estimate gDNA contamination in RNA-seq with increased accuracy, a more complicated experiment should be performed, such as adding more concentration gradients without bias toward one specific concentration. Moreover, a target intergenic region should be defined to more accurately estimate the magnitude gDNA contamination inherent in RNA-seq; and more important, to accurately estimate the proportion of gDNA contamination of any type of test material subjected to RNA-seq. To achieve this type of intergenic region, more tissue/cell types should be included to identify a comprehensive transcribed region. It follows that more accurate adjustment of gene expression levels will be achieved.

Another direct approach to adjust gene expression levels is to distinguish the reads of gDNA and reverse-transcribed cDNAs and delete gDNA reads from RNA-seq data, which is a much more difficult way to find a solution. Nowadays, extracellular RNAs (exRNAs) are emerging as potential biomarkers of disease, and gDNA contamination is a major problem to solve [9]. The experimental strategy provided here will be useful for this purpose.

Our results emphasized that analysing results of low-abundance transcripts should be carefully interpreted. In addition to the alteration in levels of low-abundance transcripts caused by gDNA contamination, the noise of RNA-seq technology hinders their accurate quantitation [4, 26]. Studies using microarray technology exclude probes with low intensities to increase the reliability of results [23, 27], because such probes may exhibit higher variances than high-intensity probes [28]. Further, RT-qPCR analysis, which is likely contaminated with gDNA, such contamination may exert more influence on the characterization of genes expressed at low levels [5]. These facts prevent the characterization of features of these genes, requiring great care in interpreting their analysis.

## Conclusions

The results of our present study fill a gap in our knowledge regarding how gDNA contamination influences the quantitation of transcriptional profiles using technologies such as RNA-seq. Further, the results of the present study support the finding that DNase does completely digest DNA, and more important, provides a strategy to estimate the residual gDNA after DNase digestion. Moreover, the proposed methods developed to correct expression data may help yield reliable results. In conclusion, we show here that gDNA contamination altered the quantitation of low-abundance transcripts. Moreover, great caution should be exercised when interpreting the results associated with such genes.

## Methods

### Cell culture

HapMap lymphoblast cell lines were purchased from the Coriell Institute. Lymphoblasts were cultured at 37 °C in RPMI 1640 medium supplemented with 15% Fetal Bovine Serum and 2 mM L-glutamate in a humidified incubator with an atmosphere of 5% CO_2_. On day 0, lymphoblasts were seeded at 200,000 cells /ml in T75 flasks (50-ml medium/flask) with loose caps and incubated in an upright position. On day 2, lymphoblasts were centrifuged at 100 g for 10 min and suspended in fresh medium. On day 4, when the lymphoblast concentration reached 600,000–800,000/ml, cells were harvested (centrifugation at 100 g for 10 min) and washed once with fresh medium.

### Genomic DNA isolation

Cell pellets (1.0 × 10^7^ cells) were resuspended in 18 ml of solution I (4.5 ml of 20% [w/v] glucose, 2.5 ml of 1 M Tris–HCl pH 8.0, 2 ml 0.5 M EDTA pH 8.0, lysozyme 2.5 g, and 91 ml of ddH_2_O). The samples incubated at room temperature for 10 min, 36 ml of ice-cold solution II (20 ml of 1 M NaOH, 10 ml of 10% SDS, 70 ml of ddH_2_O) was added to the samples with gentle inversion, and the samples were placed on ice for 10 min. Next, 27 ml of ice-cold Solution III (60 ml 5 M potassium acetate, 11.5 ml of glacial acetic acid, 28.5 ml of ddH2O) was added to the sample followed by thorough mixing. After placing in ice for 10 min, the samples were centrifuged at 11,300 g for 10 min (Beckman J2-21, JA10 rotor). The supernatant was poured through sterile cheesecloth, 50 ml of isopropanol was added, and the samples were placed on ice for 10 min. The supernatant was discarded after centrifugation at 11,300 g for 10 min (Beckman, J2-21, JA10 rotor). The residual white pellet was washed with 75% ethanol, dried, and dissolved in adding 9 ml of TE buffer (10 mM Tris–HCl pH 7.5, 1 mM EDTA pH 8.0). Cesium chloride (CsCl, Sigma-Aldrich) and ethidium bromide (10 mg/ml in TE buffer) were added to the samples, 8.5 g and 0.125 ml, respectively. The tube was then covered with foil to protect against light and centrifuged at 2,100 g for 10 min at room temperature (IEC clinical centrifuge). The supernatant was transfer to a 5/8 " × 3-" Quick Seal tube, heat-sealed and centrifuged at 447,000 g at 20 °C for 18 h (Beckman ultracentrifuge, rotor VTi 80). The DNA was visualized with UV light and placed in a 1/2" × 2" Quick Seal tube. The tube was filled with CsCl (1 g/ml in TE buffer) and then centrifuged at 645,000 g at 20 °C for 6 h (Beckman ultracentrifuge, rotor VTi 90). DNA bands were collected under UV light and washed with saturated butanol until the pink color disappeared under UV light. Next, 2.5 volumes of 100% ethanol were added to the extract followed by the addition of 0.1 volume of 5 M NaCl. The tube was then gently inverted until white strands of DNA appeared. The tube was centrifuged at 16,000 g at 4 °C for 10 min (Spectrafuge 16 M, National Labnet Co.) to collect the DNA, and the DNA pellet was washed once with 75% ethanol and dried in a speed vacuum for 2 min (Eppendorf Vacufuge, Brinkman Instruments, Inc.). The DNA pellet was resuspended in TE buffer. DNA concentrations and the 260/280 ratios were determined using a NanoDrop.

### Total RNA isolation

An RNeasy Mini Kit (250) was purchased from QIAGEN, and manufacturer’s protocol was followed with minor modification. Briefly, the pellet (1.0 × 10^7^ cells) was resuspended in 1,200 µl of RLT buffer, the lysate was further homogenized five times by passage through a blunt 20-gage, and 1,200 µl of 70% ethanol was added. The homogenized lysate was centrifuged in a RNeasy spin column at 10,000 g for 15 s, washed once with RWI buffer once and twice with RPE buffer. Total RNA was collected using two elutions with 50 µl of RNase-free H_2_O and then centrifugation at 8,000 g for 1 min. Total RNA concentrations and 260/280 ratios were measured using a NanoDrop. RINs were determined using a 2100 Bioanalyzer (Agilent). The DNase digestion was not performed during RNA isolation.

### RNA/DNA mixing, library construction and sequencing

DNA were added to and mixed with RNA after DNase treatment according to their concentrations (µg/µl) as shown in Fig. 2. RiboMinus Eukaryote Kit for RNA-seq (Invitrogen) was used to remove ribosomal RNA. Sequencing libraries were generated using this rRNA-depleted RNA using a TruSeq Stranded Total RNA Library Prep Kit. A TruSeq RNA Library Prep Kit (Illumina) was used to enrich for polyadenylated mRNA and to generate polyadenylated transcript-sequencing libraries. All procedures followed the manufacturer’s instructions. Sequencing was performed using an Illumina HiSeq 2000.

### Estimating gDNA contamination in RNA-seq samples

Genomic DNA contamination were estimated using a simple linear regression model built using the gDNA concentration as the input and the mapping ratio within the intergenic region as the outcome. The two parameters of the regression model that described library features after certain derivations are discussed below. For convenience, we derived the regression model as follows:

$${{\varvec{M}}{\varvec{a}}{\varvec{p}}{\varvec{p}}{\varvec{i}}{\varvec{n}}{\varvec{g}}\_{\varvec{r}}{\varvec{a}}{\varvec{t}}{\varvec{i}}{\varvec{o}}}_{{\varvec{I}}{\varvec{R}}}=\boldsymbol{ }\boldsymbol{\alpha }\boldsymbol{ }\cdot {\varvec{c}}\boldsymbol{ }\cdot {{\varvec{p}}}_{{\varvec{I}}{\varvec{R}}}\cdot \boldsymbol{ }{{\varvec{D}}{\varvec{N}}{\varvec{A}}}_{{\varvec{a}}}+\boldsymbol{ }\boldsymbol{\alpha }\cdot \boldsymbol{ }{\varvec{c}}\cdot \boldsymbol{ }{{\varvec{p}}}_{{\varvec{I}}{\varvec{R}}}\cdot \boldsymbol{ }{{\varvec{D}}{\varvec{N}}{\varvec{A}}}_{{\varvec{r}}}+{\varvec{\upalpha}}\cdot {{\varvec{c}}{\varvec{D}}{\varvec{N}}{\varvec{A}}}_{{\varvec{I}}{\varvec{R}}}+\boldsymbol{ }{\varvec{\varepsilon}}$$

Let ***Mapping_ratio***_***IR***_ represents the mapping ratio within the intergenic region, ***DNA***_***a***_ represents the DNA concentration of an RNA/DNA mixture, ***DNA***_***r***_ represents the residual DNA concentration in total RNA after DNase digestion, and ***cDNA***_***IR***_ represents the concentration of the cDNA produced by unannotated transcripts located within an intergenic region. The coefficient **α** describes the relation between ***mapping_ratio***_***IR***_ and DNA proportion from the intergenic region, ***c*** represents the DNA capture coefficient during library preparation, and ***p***_***IR***_ represents the proportion of the intergenic region of the complete genome.

The main assumptions employed to construct this model were as follows: 1) Mapped reads (million mapped reads per million reads, i.e., mapping ratio) of the target region are a linear function of the proportion of DNA from that region after library preparation. 2) The proportions of DNAs representing intergenic and coding regions in the added and residual DNAs are the same among all replicates and correspond to their proportions in the complete genome. 3) The efficiencies of capturing DNA during target RNA capturing steps (enrichment of polyadenylated transcripts or depletion of ribosomal RNA) are the same for intergenic and coding regions among replicates, prepared using polyadenylated mRNA enrichment and ribosomal depletion library preparation methods, respectively. A brief description of components of each step during library preparation is presented in Supplementary Figure S6, Additional File 2.

The derivation of the equation started with assumption 1 for a sequenced sample as follows:

1 $${{\varvec{m}}{\varvec{a}}{\varvec{p}}{\varvec{p}}{\varvec{e}}{\varvec{d}}\_{\varvec{r}}{\varvec{e}}{\varvec{a}}{\varvec{d}}{\varvec{s}}}_{{\varvec{t}}{\varvec{a}}{\varvec{r}}{\varvec{g}}{\varvec{e}}{\varvec{t}}}={{\varvec{m}}{\varvec{a}}{\varvec{p}}{\varvec{p}}{\varvec{i}}{\varvec{n}}{\varvec{g}}\_{\varvec{r}}{\varvec{a}}{\varvec{t}}{\varvec{i}}{\varvec{o}}}_{{\varvec{t}}{\varvec{a}}{\varvec{r}}{\varvec{g}}{\varvec{e}}{\varvec{t}}}=\left(\boldsymbol{\alpha }\cdot {{\varvec{p}}{\varvec{r}}{\varvec{o}}{\varvec{p}}{\varvec{o}}{\varvec{r}}{\varvec{t}}{\varvec{i}}{\varvec{o}}{\varvec{n}}}_{{\varvec{t}}{\varvec{a}}{\varvec{r}}{\varvec{g}}{\varvec{e}}{\varvec{t}}}+ {\varvec{\varepsilon}}\right)$$

where ***mapped_reads***_***target***_ represents the mapped reads of the target region, ***mapping_ratio***_***target***–_ represents the mapping ratio of the target region, and ***proportion***_***target***_ represents the proportion of DNA of the target region. For the intergenic region, the proportion of DNA comprises gDNA of the intergenic region, captured during target RNA capture, and cDNAs of transcripts from the intergenic region. Thus, Eq. **(****2****)** was derived from **(1)** as follows:

2 $${{\varvec{m}}{\varvec{a}}{\varvec{p}}{\varvec{p}}{\varvec{i}}{\varvec{n}}{\varvec{g}}\_{\varvec{r}}{\varvec{a}}{\varvec{t}}{\varvec{i}}{\varvec{o}}}_{IR}= \boldsymbol{\alpha }\cdot \left({{\varvec{D}}{\varvec{N}}{\varvec{A}}}_{{\varvec{c}}{\varvec{a}}{\varvec{p}}{\varvec{t}}{\varvec{u}}{\varvec{r}}{\varvec{e}}{\varvec{d}}\_{\varvec{I}}{\varvec{R}}}+{{\varvec{c}}{\varvec{D}}{\varvec{N}}{\varvec{A}}}_{{\varvec{I}}{\varvec{R}}}\right)+{\varvec{\varepsilon}}$$

where ***DNA***_***captured_IR***_ represents the proportion of DNA in the intergenic region representing the added and residual DNAs, and ***cDNA***_***IR***_ represents the proportion of DNA from the cDNA of the intergenic region.

The added and residual DNAs comprise DNAs of the intergenic and coding regions. Therefore, we calculated the total proportion of intergenic DNA according to assumption 2) as follows:

3 $${{\varvec{D}}{\varvec{N}}{\varvec{A}}}_{{\varvec{I}}{\varvec{R}}}={{\varvec{p}}}_{{\varvec{I}}{\varvec{R}}}\cdot ({{\varvec{D}}{\varvec{N}}{\varvec{A}}}_{{\varvec{a}}}+{{\varvec{D}}{\varvec{N}}{\varvec{A}}}_{{\varvec{r}}})$$

where ***DNA***_***IR***_ represents the proportion of DNA of intergenic region and ***DNA***_***a***_ and ***DNA***_***r***_ represent the proportions of the added DNA and residual DNAs, respectively.

The target RNA is captured during library preparation. DNA contamination is enriched during this step. According to assumption 3), the captured DNA of the intergenic region during the target enrichment step is represented by the equation as follows:

4 $${{\varvec{D}}{\varvec{N}}{\varvec{A}}}_{{\varvec{c}}{\varvec{a}}{\varvec{p}}{\varvec{t}}{\varvec{u}}{\varvec{r}}{\varvec{e}}{\varvec{d}}\_{\varvec{I}}{\varvec{R}}}={\varvec{c}}\cdot {{\varvec{D}}{\varvec{N}}{\varvec{A}}}_{{\varvec{I}}{\varvec{R}}}={\varvec{c}}\cdot {{\varvec{p}}}_{{\varvec{I}}{\varvec{R}}}\cdot ({{\varvec{D}}{\varvec{N}}{\varvec{A}}}_{{\varvec{a}}}+{{\varvec{D}}{\varvec{N}}{\varvec{A}}}_{{\varvec{r}}})$$

Substituting Eq. **(****4****)** into **(2)** generates the model function described above:

5 $${{\varvec{M}}{\varvec{a}}{\varvec{p}}{\varvec{p}}{\varvec{i}}{\varvec{n}}{\varvec{g}}\_{\varvec{r}}{\varvec{a}}{\varvec{t}}{\varvec{i}}{\varvec{o}}}_{{\varvec{I}}{\varvec{R}}}=\boldsymbol{\alpha } \cdot {\varvec{c}} \cdot {{\varvec{p}}}_{{\varvec{I}}{\varvec{R}}}\cdot {{\varvec{D}}{\varvec{N}}{\varvec{A}}}_{{\varvec{a}}}+ \boldsymbol{\alpha }\cdot {\varvec{c}}\cdot {{\varvec{p}}}_{{\varvec{I}}{\varvec{R}}}\cdot {{\varvec{D}}{\varvec{N}}{\varvec{A}}}_{{\varvec{r}}}+ {\varvec{\upalpha}}\cdot {{\varvec{c}}{\varvec{D}}{\varvec{N}}{\varvec{A}}}_{{\varvec{I}}{\varvec{R}}}+ {\varvec{\varepsilon}}$$

For Poly (A) Selection and Ribo-Zero:

6 $${{\varvec{M}}{\varvec{a}}{\varvec{p}}{\varvec{p}}{\varvec{i}}{\varvec{n}}{\varvec{g}}\_{\varvec{r}}{\varvec{a}}{\varvec{t}}{\varvec{i}}{\varvec{o}}}_{{\varvec{I}}{\varvec{R}}\_{\varvec{P}}{\varvec{A}}}=\boldsymbol{\alpha } \cdot {{\varvec{c}}}_{{\varvec{P}}{\varvec{A}}} \cdot {{\varvec{p}}}_{{\varvec{I}}{\varvec{R}}}\cdot {{\varvec{D}}{\varvec{N}}{\varvec{A}}}_{{\varvec{a}}}+ \boldsymbol{\alpha }\cdot {{\varvec{c}}}_{{\varvec{P}}{\varvec{A}}}\cdot {{\varvec{p}}}_{{\varvec{I}}{\varvec{R}}}\cdot {{\varvec{D}}{\varvec{N}}{\varvec{A}}}_{{\varvec{r}}}+ {\varvec{\upalpha}}\cdot {{\varvec{c}}{\varvec{D}}{\varvec{N}}{\varvec{A}}}_{{\varvec{I}}{\varvec{R}}\_{\varvec{P}}{\varvec{A}}}+ {\varvec{\varepsilon}}$$

7 $${{\varvec{M}}{\varvec{a}}{\varvec{p}}{\varvec{p}}{\varvec{i}}{\varvec{n}}{\varvec{g}}\_{\varvec{r}}{\varvec{a}}{\varvec{t}}{\varvec{i}}{\varvec{o}}}_{{\varvec{I}}{\varvec{R}}\_{\varvec{R}}{\varvec{Z}}}=\boldsymbol{\alpha } \cdot {{\varvec{c}}}_{{\varvec{R}}{\varvec{Z}}} \cdot {{\varvec{p}}}_{{\varvec{I}}{\varvec{R}}}\cdot {{\varvec{D}}{\varvec{N}}{\varvec{A}}}_{{\varvec{a}}}+ \boldsymbol{\alpha }\cdot {{\varvec{c}}}_{{\varvec{R}}{\varvec{Z}}}\cdot {{\varvec{p}}}_{{\varvec{I}}{\varvec{R}}}\cdot {{\varvec{D}}{\varvec{N}}{\varvec{A}}}_{{\varvec{r}}}+ {\varvec{\upalpha}}\cdot {{\varvec{c}}{\varvec{D}}{\varvec{N}}{\varvec{A}}}_{{\varvec{I}}{\varvec{R}}\_{\varvec{R}}{\varvec{Z}}}+ {\varvec{\varepsilon}}$$

where footnoted coefficients containing _***PA***_ and _***RZ***_ correspond to Poly (A) Selection and Ribo-Zero, respectively.

For unannotated transcripts, we simply assumed that Ribo-Zero retains all Poly (A) Selection-enriched unannotated transcripts, because the latter specifically enriches polyadenylated transcripts, and Ribo-Zero theoretically captures all transcripts. Thus, after reverse transcription, the relationship between the proportions of cDNA of the intergenic region during Ribo-Zero and Poly (A) Selection is as follows:

8 $${{\varvec{c}}{\varvec{D}}{\varvec{N}}{\varvec{A}}}_{{\varvec{I}}{\varvec{R}}\_{\varvec{R}}{\varvec{Z}}}={{\varvec{c}}{\varvec{D}}{\varvec{N}}{\varvec{A}}}_{{\varvec{I}}{\varvec{R}}\_{\varvec{P}}{\varvec{A}}}+{{\varvec{c}}{\varvec{D}}{\varvec{N}}{\varvec{A}}}_{{\varvec{I}}{\varvec{R}}\_{\varvec{R}}{\varvec{Z}}\_{\varvec{u}}{\varvec{n}}{\varvec{i}}{\varvec{q}}{\varvec{u}}{\varvec{e}}}$$

where ***cDNA***_***IR_RZ***_ and ***cDNA***_***IR_PA***_ represent the proportions of cDNA of the intergenic region of Ribo-Zero and Poly (A) Selection, respectively, and ***cDNA***_***IR_RZ_unique***_ represents proportion of unannotated transcripts unlikely captured by Poly (A) Selection.

Poly (A) Selection is less likely to contribute to DNA contamination, and it is therefore possible to first estimate ***cDNA***_***IR_PA***_ and then ***cDNA***_***IR_RZ_unique***_. The relationship between ***cDNA***_***IR_PA***_ and ***cDNA***_***IR_RZ_unique***_ is defined assuming that the relative abundances of transcripts (from coding and noncoding genes) are similar in annotated and unannotated regions. Assuming Poly (A) Selection enriches for coding genes and Ribo-Zero enriches for coding as well as noncoding genes in the intergenic region, the ratio ***cDNA***_***IR_PA***_ to ***cDNA***_***IR_RZ_unique***_ is approximately the ratio between coding and noncoding genes in the annotated region as follows:

9 $${{\varvec{c}}{\varvec{D}}{\varvec{N}}{\varvec{A}}}_{{\varvec{I}}{\varvec{R}}\_{\varvec{R}}{\varvec{Z}}\_{\varvec{u}}{\varvec{n}}{\varvec{i}}{\varvec{q}}{\varvec{u}}{\varvec{e}}}=\frac{{{\varvec{n}}}_{{\varvec{n}}{\varvec{o}}{\varvec{n}}{\varvec{c}}{\varvec{o}}{\varvec{d}}{\varvec{i}}{\varvec{n}}{\varvec{g}}}}{{{\varvec{n}}}_{{\varvec{c}}{\varvec{o}}{\varvec{d}}{\varvec{i}}{\varvec{n}}{\varvec{g}}}}\cdot {{\varvec{c}}{\varvec{D}}{\varvec{N}}{\varvec{A}}}_{{\varvec{I}}{\varvec{R}}\_{\varvec{P}}{\varvec{A}}}$$

where ***n***_***coding***_ and ***n***_***noncoding***_ represent the numbers of annotated coding and noncoding genes, respectively, in a gene transfer format (GTF) file. Substituting Eqs. **(****8****) and (****9****)** into (7) yields the equation as follows:

10 $${{\varvec{M}}{\varvec{a}}{\varvec{p}}{\varvec{p}}{\varvec{i}}{\varvec{n}}{\varvec{g}}\_{\varvec{r}}{\varvec{a}}{\varvec{t}}{\varvec{i}}{\varvec{o}}}_{{\varvec{I}}{\varvec{R}}\_{\varvec{R}}{\varvec{Z}}}=\boldsymbol{\alpha }\cdot {{\varvec{c}}}_{{\varvec{R}}{\varvec{Z}}}\cdot {{\varvec{p}}}_{{\varvec{I}}{\varvec{R}}}\cdot {{\varvec{D}}{\varvec{N}}{\varvec{A}}}_{{\varvec{a}}}+\boldsymbol{\alpha }\cdot {{\varvec{c}}}_{{\varvec{R}}{\varvec{Z}}}\cdot {{\varvec{p}}}_{{\varvec{I}}{\varvec{R}}}\cdot {{\varvec{D}}{\varvec{N}}{\varvec{A}}}_{{\varvec{r}}}+{\varvec{\upalpha}}\cdot \left(1+\frac{{{\varvec{n}}}_{{\varvec{n}}{\varvec{o}}{\varvec{n}}-{\varvec{c}}{\varvec{o}}{\varvec{d}}{\varvec{i}}{\varvec{n}}{\varvec{g}}}}{{{\varvec{n}}}_{{\varvec{c}}{\varvec{o}}{\varvec{d}}{\varvec{i}}{\varvec{n}}{\varvec{g}}}}\right)\cdot {{\varvec{c}}{\varvec{D}}{\varvec{N}}{\varvec{A}}}_{{\varvec{I}}{\varvec{R}}\_{\varvec{P}}{\varvec{A}}}+{\varvec{\varepsilon}}$$

By combining the fit determined using linear regression of Poly (A) Selection and Ribo-Zero, the residual DNA contamination in RNA-seq (***DNA***_***r***_) can be estimated.

After the linear regression model is built, the total gDNA contamination of one sequenced sample is estimated by transforming the above linear regression model as follows:

11 $${\varvec{g}}{\varvec{D}}{\varvec{N}}{\varvec{A}}=\frac{{{\varvec{m}}{\varvec{a}}{\varvec{p}}{\varvec{p}}{\varvec{i}}{\varvec{n}}{\varvec{g}}\_{\varvec{r}}{\varvec{a}}{\varvec{t}}{\varvec{i}}{\varvec{o}}}_{{\varvec{I}}{\varvec{R}}\_{\varvec{R}}{\varvec{Z}}}-{\varvec{\upalpha}}\cdot {{\varvec{c}}{\varvec{D}}{\varvec{N}}{\varvec{A}}}_{{\varvec{I}}{\varvec{R}}\_{\varvec{R}}{\varvec{Z}}}}{\boldsymbol{\alpha } \cdot {{\varvec{c}}}_{{\varvec{R}}{\varvec{Z}}} \cdot {{\varvec{p}}}_{{\varvec{I}}{\varvec{R}}}}+{\varvec{\varepsilon}}$$

where ***gDNA*** corresponds to total gDNA contamination.

### Sequencing data quality control and trimming

FastQC [29] and FastQScreen [30] were used to evaluate the quality of sequencing data and to identify potential contamination of RNA-seq. Trimmomatic [31] were used for trimming and filtering reads. Parameters of tools used in this section were provided in Additional File 3.

### Quantitation of gene expression and the intergenic region

HISAT2, StringTie, and Ballgown pipeline [32] were used to map reads to the human genome and to quantify gene expression. The reference genome GRCh38 (version 84) and the gene annotation file (GTF format) were downloaded from GENCODE (version 22). We used FPKM to normalize gene expression levels, and a constant = 0.01 was added to gene expression levels of all samples before further downstream analysis. Genes with expression levels < 0.02 in 30% of samples were excluded for Poly (A) Selection and Ribo-Zero. The Student *t* test was used to identify DEGs. A gene was considered a DEG with unadjusted *p* < 0.05 and absolute value of log2(fold-change) > 1. Correlation tests based on the Pearson correlation in R [33] were used to determine if the expression levels of a gene correlated with gDNA concentrations (*p* < 0.05, two-sided, Bonferroni adjusted).

The intergenic regions were defined as genomic regions not overlapped by annotated genes or newly identified annotated transcripts in all libraries of Poly (A) Selection and libraries of Ribo-Zero with 0% DNA. New transcripts were identified and merged using StringTie using the reference gene annotation file. BEDTools [34] were used to generate the bed file of intergenic regions. SAMtools [35] was used to count the reads mapped to intergenic regions and total mapped reads. The mapping ratio of intergenic regions was then calculated. Parameters of tools used in this section were provided in Additional File 3.

### KEGG pathway enrichment analysis

DEGs were subjected to KEGG pathway enrichment analysis using enrichKEGG() function and default parameters of the R package clusterProfiler [36].

### Adjusting gene expression according to reads mapped within the intergenic region

To adjust gene expression levels, the value of genomic DNA should be discarded from the total. Thus, specific transcription of one gene is calculated as follows:

12 $${{\varvec{F}}{\varvec{P}}{\varvec{K}}{\varvec{M}}}_{{\varvec{R}}{\varvec{N}}{\varvec{A}}}={{\varvec{F}}{\varvec{P}}{\varvec{K}}{\varvec{M}}}_{{\varvec{t}}{\varvec{o}}{\varvec{t}}{\varvec{a}}{\varvec{l}}}-{{\varvec{F}}{\varvec{P}}{\varvec{K}}{\varvec{M}}}_{{\varvec{D}}{\varvec{N}}{\varvec{A}}}$$

where ***FPKM***_***DNA***_ and ***FPKM***_***RNA***_ represent the expression level values of gDNA and RNA, and ***FPKM***_***total***_ represents the expression value calculated using quantitation software. Here, the expression values were given by Ballgown.

According to the definition of FPKM, the expression of a single gene in contaminated DNA is calculated as follows:

13 $${{\varvec{F}}{\varvec{P}}{\varvec{K}}{\varvec{M}}}_{{\varvec{D}}{\varvec{N}}{\varvec{A}}}=\frac{{{\varvec{m}}{\varvec{a}}{\varvec{p}}{\varvec{p}}{\varvec{e}}{\varvec{d}}\_{\varvec{f}}{\varvec{r}}{\varvec{a}}{\varvec{g}}{\varvec{m}}{\varvec{e}}{\varvec{n}}{\varvec{t}}}_{{\varvec{D}}{\varvec{N}}{\varvec{A}}}}{{{\varvec{t}}{\varvec{o}}{\varvec{t}}{\varvec{a}}{\varvec{l}}\_{\varvec{r}}{\varvec{e}}{\varvec{a}}{\varvec{d}}{\varvec{s}}}_{{\varvec{M}} }\cdot {{\varvec{g}}{\varvec{e}}{\varvec{n}}{\varvec{e}}\_{\varvec{l}}{\varvec{e}}{\varvec{n}}}_{{\varvec{k}}{\varvec{b}}}}$$

where ***mapped_fragment***_***DNA***_ represents the number of mapped fragments generated from DNA contamination, ***total_reads***_***M***_ represents the number of reads mapped to the genome per million, and ***gene_len***_***kb***_ represents 1 gene/kb.

Assuming that reads derived from DNA contamination are uniformly distributed throughout the genome, the fragments originating from DNA contamination mapped to the intergenic region, as well as to a specific gene, are therefore identical. Thus, ***FPKM***_***DNA***_ of one specific gene can be estimated by estimating ***FPKM***_***DNA***_ of the intergenic region as follows:

14 $${{\varvec{F}}{\varvec{P}}{\varvec{K}}{\varvec{M}}}_{{\varvec{D}}{\varvec{N}}{\varvec{A}}\_{\varvec{g}}{\varvec{e}}{\varvec{n}}}={{\varvec{F}}{\varvec{P}}{\varvec{K}}{\varvec{M}}}_{{\varvec{D}}{\varvec{N}}{\varvec{A}}\_{\varvec{I}}{\varvec{R}}}=\frac{{{\varvec{m}}{\varvec{a}}{\varvec{p}}{\varvec{p}}{\varvec{e}}{\varvec{d}}\_{\varvec{f}}{\varvec{r}}{\varvec{a}}{\varvec{g}}{\varvec{m}}{\varvec{e}}{\varvec{n}}{\varvec{t}}}_{{\varvec{D}}{\varvec{N}}{\varvec{A}}\_{\varvec{I}}{\varvec{R}}}}{{{\varvec{t}}{\varvec{o}}{\varvec{t}}{\varvec{a}}{\varvec{l}}\_{\varvec{r}}{\varvec{e}}{\varvec{a}}{\varvec{d}}{\varvec{s}}}_{{\varvec{M}} }\cdot { {\varvec{i}}{\varvec{n}}{\varvec{t}}{\varvec{e}}{\varvec{r}}{\varvec{g}}{\varvec{e}}{\varvec{n}}{\varvec{i}}{\varvec{c}}\_{\varvec{l}}{\varvec{e}}{\varvec{n}}}_{{\varvec{k}}{\varvec{b}}}}$$

where ***FPKM***_***DNA_gen***_ and ***FPKM***_***DNA_IR***_ represent the expression values specific to contamination with DNA of a specific gene and of an intergenic region, ***mapped_fragment***_***DNA_IR***_ represents the number of mapped fragments originating from DNA contamination of the intergenic region, and ***intergenic_len***_***kb***_ is the length of the intergenic region per kilobase.

If the newly identified unannotated transcripts are excluded from the intergenic region, the mapped fragments of the intergenic region should be identical to the mapped fragments originating from DNA contamination of the intergenic region. Thus, using mapped fragments within the intergenic region, the expression value is adjusted by subtracting ***FPKM***_***DNA_gen***_ from ***FPKM***_***total***_. SAMtools [35] was used to extract fragments mapped to the intergenic region according to the HISAT2 mapping results.

### Statistical analysis

Statistical analysis was performed using R [33]. The functions hclust() and pca() were used to for HCA and PCA. Statistical analyses were performed using the functions cor.test(), fisher.test(), and t.test(); Fisher’s exact test; and the Student *t* test. The GC content of each gene was determined using the biomaRt package [37]. The scripts that reproduce all the steps of this study are available on GitHub (https://github.com/HaiGenBuShang/Genomic_DNA_in_RNA_seq).

## Supplementary Information

**Additional file 1.**

**Additional file 2.**

**Additional file 3.**

## Data Availability

The datasets generated and/or analysed during the current study are available in the Genome Sequence Archive for Human of National Genomics Data Center repository, [accession number HRA001834].

## Abbreviations

gDNA: Genomic DNA
DEG: Differentially Expressed Gene
RT-qPCR: Reverse transcription quantitative PCR (RT-qPCR)
SEQC: Sequencing Quality Control
exRNA-seq: Extracellular RNA Sequencing
lncRNA: Long Non-coding RNA
FFPE: Formalin fixed–paraffin embedded
FPKM: Fragments per Kilobase of Transcript per Million Read Pairs
KEGG: Kyoto encyclopedia of genes and genomes
HCA: Hierarchical cluster analysis
PCA: Principal component analysis
GTF: Gene Transfer Format
