# Supplementary material for: Genes expressed at low levels raise false discovery rates in RNA samples contaminated with genomic DNA
Source: BMC Genomics. 2022 Aug 3;23:554. doi: 10.1186/s12864-022-08785-1 (PMC9351092; doi:10.1186/s12864-022-08785-1)
Supplement: Supplementary file 1 — Additional file 1. [file 12864_2022_8785_MOESM1_ESM.docx]

Table S1: Regression coefficients of gDNA contamination and mapping ratio of the intergenic region of Poly (A) Selection

| Variable | B | SE B | β | t-statistic | p-value |
| --- | --- | --- | --- | --- | --- |
| Constant | 0.0117 | 7.08e-05 | 0.000 | 164.613 | <0.001 |
| gDNA concentration | −1.52e-03 | 1.73e-03 | −0.238 | −0.882 | 0.394 |

R^2^ = 0.0565

F(1, 13) = 0.778, p = 0.394

Table S2: Regression coefficients of gDNA contamination and mapping ratio of the intergenic region of Ribo-Zero

| Variable | B | SE B | β | t-statistic | p-value |
| --- | --- | --- | --- | --- | --- |
| Constant | 0.0472 | 1.73e-03 | 0.000 | 27.247 | <0.001 |
| gDNA concentration | 0.658 | 0.0423 | 0.974 | 15.542 | <0.001 |

R^2^ = 0.949

F(1, 13) = 241.568, p = 8.90e-10
