# Supplementary material for: Genes expressed at low levels raise false discovery rates in RNA samples contaminated with genomic DNA
Source: BMC Genomics. 2022 Aug 3;23:554. doi: 10.1186/s12864-022-08785-1 (PMC9351092; doi:10.1186/s12864-022-08785-1)
Supplement: Supplementary file 2 — Additional file 2. [file 12864_2022_8785_MOESM2_ESM.docx]

**Figure S1.** Most genes detected in association with gDNA were expressed at lower levels. Distribution of expression levels of genes correlated with gDNA in Ribo-Zero. The x-axis represents expression (log2(FPKM)), and the y-axis represents density. Five hundred and two genes correlated with gDNA in Ribo-Zero and Poly (A) Selection, respectively.

**Figure S2.** The number of DEGs increased as a function of increasing gDNA concentration in Ribo-Zero, and remained approximately constant in Poly (A) Selection. Numbers of DEGs of Poly (A) Selection and Ribo-Zero at different gDNA concentrations. Each bar represents a different treatment, the y-axis represents the number of DEG between libraries with > 0% (Treatment) and with 0% (Control) gDNA. The purple and yellow bars represent Poly (A) Selection and Ribo-Zero.

**Figure S3.** Numerous background DEGs significantly altered adjusted p values. a) Numbers of DEGs numbers in the background of pathway analysis between Ribo-Zero libraries (0% to 10% gDNA) and Poly (A) Selection libraries (0% gDNA) and between Ribo-Zero libraries with 10% and with 0% gDNA. b) Venn diagram of DEGs in “hsa04740”, the most enriched pathway in Ribo-Zero libraries, between Ribo-Zero libraries with 10% gDNA and Poly (A) Selection libraries with 0% gDNA and between Ribo-Zero libraries with 10% and with 0% gDNA. Most DEGs between Ribo-Zero libraries were detected in the comparison of Poly (A) Selection and Ribo-Zero. c) The most enriched pathway “hsa04740” in Ribo-Zero libraires was not significantly enriched compared with Ribo-Zero and Poly (A) Selection libraries with 10% and 0% gDNA. The large number of DEGs in “PA VS RZ” contributed to a higher p-value compared with “RZ”. d) The p value indicated statistical significance when the number of DEGs in the background decreased to approximately 1650 for “hsa04740” for “PA VS RZ” (Fisher exact test, FDR adjusted). PA: Poly (A) Selection; RZ: Ribo-Zero.

**Figure S4.** Adjusting expression levels did not reduce the number of DEGs of Poly (A) Selection libraries. DEGs were detected by comparing the treatments with gDNA contamination (Treatment) and that with 0% gDNA contamination (Control) for Poly (A) Selection libraries. The red bar represents DEGs detected before expression adjustment, and the blue bar represents DEGs detected after expression adjustment. The x-axis represents different treatments, and the y-axis represents the number of DEGs in each comparison. (*t* test; two-sided p <0.05 and |log2(fold-change)|>1).

**Figure S5.** Varying gene expression filter thresholds avoid DEGs caused by gDNA contamination. a, b) The number of overall DEGs decreased when the low-abundance filtering threshold (raw FPKM) increased at different concentrations of contaminating gDNA. a, b) Ribo-Zero and Poly (A) Selection, respectively. The x-axis represents the low-abundance gene filtering threshold, the y-axis represents the number of DEGs.

**Figure S6.** Library Preparation. Library preparation started from extracting RNA from lymphoblasts. After DNase digestion, all RNA species and residual gDNA were present in total RNA. Next, different concentrations of extracted gDNA were added to total RNA. During the next target-RNA capturing step, target RNA and some gDNA are captured. However, capture efficiencies differed for Poly (A) Selection and Ribo-Zero. In the next step, the captured RNA was reverse transcribed, and the cDNA served as template for Polymerase Chain Reaction (PCR) assays with captured DNA to generate sequencing libraries. The cDNAs and captured DNA contained DNA located in intergenic and coding regions. The cDNA sequences located in the intergenic region represents unannotated transcripts.
