# Supplementary material for: Genes expressed at low levels raise false discovery rates in RNA samples contaminated with genomic DNA
Source: BMC Genomics. 2022 Aug 3;23:554. doi: 10.1186/s12864-022-08785-1 (PMC9351092; doi:10.1186/s12864-022-08785-1)
Supplement: Supplementary file 3 — Additional file 3. [file 12864_2022_8785_MOESM3_ESM.docx]

Commands and Parameters of Used tools

Tools used in “Sequencing data quality control and trimming”

FastQC:

fastqc fastqfile_1 fastqfile_2 ...

FastQ Screen: (aligner: bowtie2)

fastq_screen --conf /path/to/FastQ_Screen/configure fastqfile

Trimmmatic:

java **-Xmx4g -jar** /path/to/trimmomatic_jar_file PE -threads 4 fastqfile_1 fastqfile_2 fastqfile_1.trimmed.fastq.gz fastqfile_1.unpaired.fastq.gz fastqfile_2.trimmed.fastq.gz fastqfile_2.unpaired.fastq.gz **ILLUMINACLIP:/path/to/adapter/file:2:30:10 LEADING:10 TRAILING:10 HEADCROP:10 SLIDINGWINDOW:4:15 MINLEN:36**

Tools used in “Quantitation of gene expression and the intergenic region”

HISAT2: (default with alignment option: **--n-ceil L,0,0.15**, **--ignore-quals**, **--nofw**, **--norc**)

hisat2 **-q -p 4 -x** /HISAT2/Reference/Index -1 fastqfile_1.trimmed.fastq.gz -2 fastqfile _2.trimmed.fastq.gz **-S** fastqfile.sam

SAMtools:

samtools view **-b** fastqfile.sam > fastqfile.bam

samtools sort **-@ 3** fastqfile.bam > fastqfile.sorted.bam

samtools view **-L** intergenic_region_bed -h fastqfile.sorted.bam | grep **-P '^\@|NH:i:1\b'** | samtools view **-b** > fastqfile.sorted.intergenic.unique.bam

samtools stats fastqfile.sorted.bam > fastqfile.stats_contains_total_mapped_reads.txt

samtools stats fastqfile.sorted.intergenic.unique.bam > fastqfile.stats_contains_intergenic_mapped_reads.txt

BEDTools:

bedtools complement **-i** transcript_locations_in_merged_gtf **-g** /chromosome/length/file > intergenic_region_bed

StringTie:

stringtie fastqfile.sorted.bam **-p 3 -a 10 -m 200 -g 50 -c 2.5 -j 1 -l STRG -M 0.95 -G** /Reference/gtf/file **-e -o** fastqfile.transcripts.stringtie.gtf **-A** fastqfile.gene.abundance.txt **-b** /Ballgown_folder/fastqfile

stringtie **--merge -G** /Reference/gtf/file **-o** merged_gtf gtf_to_be_merged_list

Ballgown:

ballgown(**dataDir = Ballgown_folder, samplePattern = ".*", meas = "FPKM"**)

Detailed analysis can be found on GitHub (https://github.com/HaiGenBuShang/Genomic_DNA_in_RNA_seq).
